# Acceptability and feasibility of a national essential medicines list in Canada: a qualitative study of perceptions of decision-makers and policy stakeholders

**DOI:** 10.1503/cmaj.190567

**Published:** 2019-10-07

**Authors:** Jordan D. Jarvis, Adrianna Murphy, Pablo Perel, Nav Persaud

**Affiliations:** London School of Hygiene & Tropical Medicine (Jarvis), London, UK; Centre for Urban Health Solution (Jarvis), St. Michael’s Hospital, Toronto, Ont.; Department of Health Services Research and Policy (Murphy); Epidemiology and Population Health Faculty (Perel), London School of Hygiene & Tropical Medicine, London, UK; Department of Family and Community Medicine (Persaud), University of Toronto, Toronto, Ont.

## Abstract

**BACKGROUND:**

Policy approaches have been considered to address inconsistent and inequitable prescription drug coverage in Canada, including a national essential medicines list. We sought to explore key factors influencing the acceptability and feasibility of an essential medicines list in Canada.

**METHODS:**

We conducted semi-structured interviews with decision-makers and other key stakeholders from government or pan-Canadian institutions, civil society and the private sector across Canada. We analyzed data using inductive thematic analysis and by applying Kingdon’s Multiple Streams Framework to analyze the emergent themes deductively.

**RESULTS:**

We conducted 21 interviews before thematic saturation was achieved. We categorized emergent themes to describe the problem, the essential medicines list policy (including content and process), and politics. There was consensus among participants that prescription drug coverage was an important problem to address. Participants differed in their views on how to define essential medicines and concerns about what would be excluded from an essential medicines list. There was consensus on important features for a process to develop an essential medicines list: an independent decision-making body, use of defined selection criteria based on quality evidence, and clear communication of the purpose of the essential medicines list. Federal government financing and the broader pharmacare model, engagement of various interest groups and changing political agendas emerged as core political factors to consider if developing a Canadian essential medicines list.

**INTERPRETATION:**

Although stakeholders’ views on the content of a Canadian essential medicines list varied, there was consensus on the process to formulate and implement an essential medicines list or common national formulary, including choosing medicines based on best evidence. Greater understanding is now needed on how patients, clinicians and the public perceive the concept of an essential medicines list.

Through Canada’s complex system of prescription drug coverage, about 21% of Canadians are covered by public provincial or territorial drug plans, 3% by federal public coverage and 70% by full or partial private insurance.^1^ An estimated 20% of Canadians are currently uninsured or underinsured.^2^,^3^ Many have detailed the failings of this system of prescription drug coverage to deliver equitable access to medicines across the country, its cost and administrative inefficiencies, sustainability concerns, and challenges related to rational use and inappropriate prescribing.^1^–^7^ Provincial, territorial and federal governments currently administer public drug plans with varied approaches to coverage, lists of covered or reimbursed drugs and formularies.^1^,^5^ The medicines included on different public formularies overlap substantially.^8^

The recent final report of the Federal Advisory Council on the Implementation of National Pharmacare, the 2019 federal budget and various other government reports have highlighted the need for a common national formulary to harmonize drug coverage across the country in the context of pharmacare policy change.^1^,^9^,^10^ A national essential medicines list has been considered as a potential policy tool to reduce variabilities in prioritized medicines across the country, guide improved prescribing, ensure quality and safety of care, and improve efficiency of medicine spending.^11^–^13^ The report of the Federal Advisory Council on the Implementation of National Pharmacare recommends that “federal, provincial and territorial governments launch national pharmacare by offering universal coverage for a list of essential medicines” as a basis for a national formulary that would set a minimum level of coverage across Canada.^10^ Universal public coverage of an essential medicines list in Canada was estimated to result in $4.27 billion annual savings and cover most of the current medication needs in the country.^14^

Although national essential medicines lists have mostly been developed in low-and middle-income countries, at least 21 high-income countries have essential medicines lists to carefully select medications that “satisfy the priority health care needs of the population,” such as the “Wise List” in Sweden.^15^–^18^ The World Health Organization (WHO) Model List of Essential Medicines serves as an international guide to help policy-makers set their national medicine priorities in their unique context.^18^

The perceptions of decision-makers and policy stakeholders about using an essential medicines list approach in Canada are not well understood. Various groups, including the pharmaceutical industry, have voiced concerns that an essential medicines approach could restrict choice or access to innovative medicines.^19^ We sought to explore the perspectives of decision-makers and other key stakeholders on a possible national essential medicines list in Canada and to identify factors influencing the acceptability and feasibility of such a policy during an important pharmacare policy window using a qualitative study.

## Methods

### Data collection

**Figure 1:**
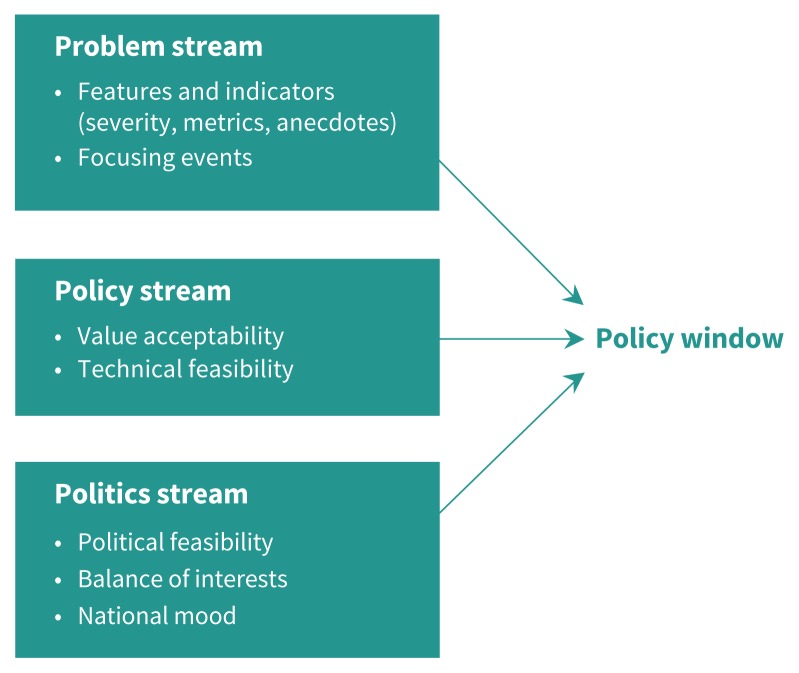
Kingdon’s Multiple Streams Framework (adapted, with permission, from Jones et al.^21^). The problem stream involves policy issues that diverse stakeholders pay attention to and desire action on. The policy stream describes viable policy solutions or instruments to solve policy problems. The politics stream describes decision-maker motives and opportunities to advance policy, influenced by factors such as interest groups and perceived political feasibility.^20^

Semistructured interviews were conducted by one author (J.D.J.) in English. We conducted interviews between July and September of 2018. The topic guide for our interviews (Appendix 1, available at www.cmaj.ca/lookup/suppl/doi:10.1503/cmaj.190567/-/DC1) was informed by Kingdon’s Multiple Streams Framework,^20^,^21^ which depicts policy change as occurring when 3 streams — problems, policies and politics *—* come together at junctures termed “policy windows” (Figure 1; supplementary description in Appendix 2, available at www.cmaj.ca/lookup/suppl/doi:10.1503/cmaj.190567/-/DC1), as well as by data-collection tools used in research on essential medicines lists in Australia and Sweden.^16^,^22^

A draft topic guide was shared with 4 experts on medicine priority-setting, both in Canada and at WHO, and refined based on their feedback. The topic guide included open-ended questions that addressed interview participants’ understanding of an essential medicines list, important processes for implementing an essential medicines list, and the roles of diverse stakeholders in this process (Appendix 1). The guide was used to prompt discussion, but interviews allowed for exploration of individual participants’ perspectives and for unexpected themes to emerge.^23^ We conducted one pilot interview and included it in the analysis, as no changes were made to the topic guide thereafter.

Our approach to sampling participants was also informed by the Multiple Streams Framework,^20^ which emphasizes the complexity of policy-making shaped by diverse stakeholders at different levels of government and outside of government. Thus, we purposively sampled key stakeholders in pharmaceutical policy with attention to Canada-wide representation from each of the following categories: federal government and pan-Canadian organizations, provincial and territorial government, civil society and the private sector.^24^,^25^ Eligibility criteria for each category are shown in Table 1. Initial participants were identified using public documents and advice from experts; subsequent participants were identified through snowball sampling, whereby recruited participants were asked to suggest other eligible participants.^25^ Our final sample size was determined by reaching thematic saturation in data collection.^26^

**Table 1:** Participant eligibility and participation

Participant category	Eligibility criteria	Participants invited	
		Interviewed	Declined or did not reply
Federal government or pan-Canadian institutions	Senior officials involved in decision-making related to prescription drug coverage, serving in federal departments or pan-Canadian agencies	5	4
Provincial or territorial government	Senior officials in positions of decision-making across diverse provincial and territorial authorities in Canada; portfolio includes prescription drug coverage	5	9
Civil society	Active nongovernmental stakeholders in national pharmacare discussions with public health mandate; senior representatives of health care professional bodies, nongovernmental organizations, unions; academics	9	3
Private sector	Senior representatives of private industry organizations that appear influential in national pharmacare discussions	2	2

No previous relationships existed between the interviewer and any of the participants. We obtained written informed consent from all participants after discussing the purpose of the research (Appendix 3, available at www.cmaj.ca/lookup/suppl/doi:10.1503/cmaj.190567/-/DC1). Where consent was not given for audio-recording, the interviewer took detailed field notes that included contextual information and nonverbal cues to incorporate into data analysis.^27^ One author (J.D.J.) and an assistant transcribed all audio-recorded interviews verbatim. We deidentified and checked transcripts and field notes for accuracy.

### Data analysis

We used inductive thematic analysis, in which only J.D.J. coded for emerging themes and concepts using NVivo 12, allowing themes to emerge from the data without a theoretical framework, as described by Braun and Clarke.^28^ Codes and themes were then reviewed by A.M., N.P. and a research assistant to enhance interrater reliability and analytic credibility.^28^ We discussed the emerging themes in relation to the research question and the existing literature, whereby a continuous consultative approach between authors promoted reflexivity and enabled deep exploration of the themes.^29^ Negative cases were investigated and discussed between all authors to test these themes and consider why they were different.^30^

After developing a coding framework, J.D.J. conducted deductive analysis using the Multiple Streams Framework,^20^ whereby the framework’s 3 established categories and considerations, such as value acceptability and technical feasibility within the policy stream (Figure 1), served to organize the analysis and theoretical comparison of inductive themes.^21^ The deductive analysis and findings were reviewed by A.M., P.P. and N.P. Disagreements in interpretation of the themes or findings were discussed among the authors to arrive at a shared interpretation. Considered in the reflexive approach were researchers’ characteristics that included our diverse experiences in pharmaceutical policy, patient care and health systems research in Canada and internationally.^29^ When designing the study and interpreting the results, we considered and tried to balance with alternative views the following perspectives: J.D.J. and N.P. have collaborated in the past with the WHO Essential Medicines List secretariat, and N.P. is studying and has advocated for an essential medicines list approach in Canada. Finally, we shared the research findings with 2 study participants who reviewed and validated the findings based on their inputs into the study.^31^ We followed the Consolidated Criteria for Reporting Qualitative Research guidelines.^24^

### Ethics approval

We obtained ethics approval through the London School of Hygiene & Tropical Medicine Ethics Committee (Ref: 15365).

## Results

### Participants

We conducted a total of 21 interviews (Table 1). J.D.J., A.M. and N.P. continuously reviewed emerging themes and agreed that saturation was reached at this point.^26^ We sent email invitations for interviews to 39 individuals across 13 provinces and territories. Five people did not respond to the request for interview, and 13 declined because of perceived conflicts of interest or time constraints; 3 of these 13 referred a colleague. Seventeen interviews were conducted over the phone and 4 in person, lasting 40–80 minutes each. Written consent was obtained for audio-recording interviews from 18 participants. Participants represented 6 diverse provinces and territories, including 5 diverse provincial or territorial governments. Nine participants were women and 12 were men.

Themes identified fell under 3 categories: perceptions of the problem, perceptions of the essential medicines list as a policy solution and the politics of an essential medicines list. Table 2 summarizes factors identified influencing acceptability and feasibility of an essential medicines list, along with quotes.

**Table 2:** Perceived factors influencing the acceptability and feasibility of an essential medicines list in Canada through the lens of Kingdon’s Multiple Streams Framework

Stream	Factors identified	Example quotes
Perceptions of the problem (problem stream)	Inequitable access to medicines across Canada	“I think number one, that those challenges [to providing prescription drugs] probably look differently depending on which province you’re in, right. That’s one of the challenges of who we cover, how we cover, and in some cases what we cover look different.” — PT5
	Health system sustainability concerns (including high medicine costs)	“Canada pays among the highest prices for medicines in the world. ... And Canada’s gotta equip itself with the institutional capacity to push back and to make sure our prices remain reasonable. And in some sense the most clinically and economically rational way of doing that is through the careful evidence-based negotiation of what gets covered and at what price is it covered.” — CSO1
		“... there’s a huge amount of inefficiency for the sake of jurisdictional autonomy.” — FED4
Policy content and process factors (policy stream)	**Content**	
	No shared understanding of an essential medicines list and how to define essential medicines	“I don’t think the public understands. And I use public not only the person on the street, but other sort of stakeholders in the health care system. I don’t think they have an agreement of what that list means, and even I don’t know if I know what that means.” — PT5
		“To me that is a policy approach that is used more for developing countries and is potential confusing term in developed countries like Canada.” — PT4
		Examples of the 2 most common ways that essential medicines were defined: “the shortest possible list of critical medicines that are needed in a jurisdiction in order to meet the primary, common and serious health needs of the population.” — CSO7 “Until you’re sick, you really do not know what you would consider essential. When you’re sick, everything is essential that you need to get better.” — CSO9
	Concerns around therapies that would be excluded from the essential medicines list	“Often anything taken away is a bad thing. Even if it was causing harm, but if one person benefited, they will see it as a bad thing. If the new list is much smaller and less, it will be seen as less benefit for the person.” — FED2
		“So what happens to those other ones, right? All the people using those. So the tension that that would create to maintain an essential drug list ... everyone will want to be deemed essential, right?” — PT4
	**Process**	
	Need for an independent and accountable decision-making body	“If it’s going to be national, some sort of pan-Canadian body or bodies that report into one central mechanism. And it would need representatives from clinical community, patients, caregivers, family members, public, methodologists — the people who will do evidence synthesis, systematic reviews, pharmacists ... and we would need people who were representing jurisdictions, so the drug plan managers. But again, not political people. And likely also specific representation from the First Nations community. ... And they should have very clear terms of reference, publicly announced meetings, you know whether their deliberations should become public. ... you would need to make sure that everybody engaged would disclose conflicts of interest and those are managed appropriately, which could mean excluding anybody with conflicts. I would say ... that there would not be reps from industry on this [decision-making body].” — FED5
	Clear and agreeable selection factors for the development and management of a list: clinical evidence and cost-effectiveness	“... if it’s truly marginally beneficial, why the heck is someone even considering to pay for it today, whether it’s public or private?” — IND1 “It’s not all or nothing, but the degree to which you fund a drug, and how much of that drug you fund, depends on how much evidence you have for benefit for that population.” — FED4
		“... it’s an exercise about trying to make good judgments about what investment in which drugs at any particular point in time will provide the best outcome for the patient. And at what cost should that occur? At what cost should that investment come.” — CSO8
	Framing and communication of an essential medicines list	“This is not about restricting access to medicines for people, it’s about improving access to medicines. There are a lot of people who do not have access to the meds that would be on an essential medicines list at all. So thinking about how you frame it I think also matters.” — CSO7
		“... you have to put it into a language where we will guarantee Canadians that the prescription drugs that will be put on the list will be scientifically proven, evidence based ... and efficient, and better prescribing habits. ... It doesn’t matter if it’s 2 drugs or 10 000, but what’s on that list is what you will need based on what the experts are saying and that’s that guarantee we need to give.” — CSO6
Political factors (politics stream)	Federal financing and the pharmacare model	“[Federal financing for] perhaps a half of the costs of that formulary, which is sort of the medicare bargain: the feds pay half, the provinces pay half, at the outset of Canadian medicare.” — CSO1
		“[Who should decide] depends on who is paying the bills. And that’s the real challenge right now, is that you have all these different payers, right. If one group actually wants to take that on, then they would be accountable for that process. ... but we would have [to] rely on a national approach that actually looks at formulary management besides just the drugs that are being submitted [to CADTH]. ... And if it is decentralized, then the jurisdictions should have the right to manage their own formulary.” — PT4
	Management of diverse stakeholder interests and safeguards against conflicts of interest by an independent body	“You also absolutely need to insulate the people involved in making the final decisions about what’s on and what’s off from political pressures. And I believe firmly that’s a benefit to our political leaders, because they are vulnerable as representatives of particular constituents ... to threats by industry stakeholders that they will withhold funding, they will withhold industrial projects, they will withhold research activities, they will lay off people working in their workforces, if a decision doesn’t go their way. ... It’s a form of political extortion, right.” — CSO1
		“I think it’s critical to have citizen involvement. I don’t think that these things should be done just by so-called experts. How you select the citizens who get involved in that, that they don’t have a particular bone to pick or a particular disease that they care about, is always very tricky. But it’s not an excuse not to have citizen involvement.” — CSO7

Note: CADTH = Canadian Agency for Drugs and Technologies in Health, CSO = civil society, FED = federal government or pan-Canadian institutions, IND = private sector, PT = provincial or territorial government.

### Perceptions of the problem

There was consensus on a problem: the prescription-medication needs of all Canadians are not being adequately met. Respondents’ views on the most important features of the problem differed. The features most frequently cited were inequitable access to medicines, high drug prices and health system inefficiencies. There was consensus on the need to address current inequitable access to medicines and variations in medication affordability across the country. The second key challenge that arose was access to affordable medicines as a key component of a more sustainable and efficient health care delivery system.

### Policy content and process factors

Five themes emerged across the content and process categories that may affect acceptability and feasibility of an essential medicines list in Canada.

#### No shared understanding or definition of essential medicines or an essential medicines list

Some did not view an essential medicines list as applicable in the Canadian context, owing to a perceived difficulty in agreeing on a set of medicines that would sufficiently cover the needs of all Canadians or owing to an association of the concept with WHO and lower-income countries. Those who voiced a more global view of essential medicines voiced less opposition to an essential medicines list and saw this idea as synonymous with a formulary or as a “specific kind of formulary” (FED4). Many respondents suggested that the use of another term to replace “essential medicines” might result in more support for the concept. The most common views articulated on what essential medicines meant was either medicines most commonly required by the population or those medically required by a given individual.

#### Concerns about what is not listed on an essential medicines list

Concerns about what would be excluded from an essential medicines list were voiced across all participant categories. Stakeholders voiced their perceptions of the public’s response to an essential medicines list either stating that “people don’t want their choice restricted” (FED3) or they described perceived opposition from specific stakeholders, namely, rare-disease patient groups, pharmaceutical and insurance industries, or certain clinicians. Others perceived that an essential medicines approach might result in coverage being taken away from some Canadians under their current private or public plans or that it would be “very restrictive*”* (IND2).

Most strongly emphasized by respondents from provincial and territorial governments and industry, and by some from civil society, was that an essential medicines list might not offer a sufficiently dynamic approach to address “the big elephant in the room” (PT5) of how to handle access to high-cost, specialized or novel therapeutics. In particular, provincial and territorial decision-makers noted concerns about “creating 2 tiers by creating an essential medicines list, because every province is still gonna struggle with how to cover what’s not on the list” (PT2).

#### An independent and accountable decision-making body

There was strong consensus around the need for an independent decision-making body, with frequent mention by interviewees of a multidisciplinary and multistakeholder “arm’s-length” agency and a committee (or committees) that would oversee the management and medicine-listing decisions of an essential medicines list. Key stakeholders to involve in the process were considered to be clinicians, patients and the public, provincial and territorial drug-plan representatives, payers (primarily governments) and experts *“*who can evaluate the evidence” (FED3). However, many also noted the tension between engagement and efficiency: “sometimes you weigh yourself way too down by having too many fingers in the pot” (PT5).

#### Core selection criteria for an essential medicines list

There was consensus among interviewees on selection factors that could serve as core, transparent criteria for selecting medicines to be included in an essential medicines list. First, that decisions be based on the best available evidence for clinical and health outcomes above all other considerations, particularly for drugs for which decision-making might be difficult in the face of limited or contested evidence. Second, that cost-effectiveness and value for money must be considered. A shared priority was to maintain a sustainable model that carefully weighs the evidence and added value of high-cost therapeutics. Many concerns existed around money currently spent on products in which the evidence is “complete crap*”* (CSO2). Several participants noted the need to evaluate societal benefit that interventions offer and to better understand opportunity costs incurred, which were a particular concern for government respondents.

#### Framing and communicating to the public and decision-makers

How an essential medicines list is framed and communicated to the public, clinicians, pharmacists and decision-makers was voiced as a key factor influencing the acceptability of an essential medicines list. Many interviewees remarked that an essential medicines list would be more acceptable to important stakeholders if it were clearly emphasized as a means to improve equitable access to evidence-based, high-quality and affordable medicines. The difficulty communicating the idea effectively, given the diverse understandings and ideas around what essential medicines and an essential medicines list mean, was acknowledged. Nevertheless, several participants were optimistic that most Canadians would respond favourably to clear communication on an essential medicines list.

### Political factors

There was consensus among participants that federal financing would be necessary to formulate, implement and sustain an essential medicines list. Provincial and territorial government respondents considered that final decisions related to medicines publicly covered should rest with the government that is paying, many seeing decision-making power as something that would likely remain with provincial and territorial governments. Possible pharmacare models were mostly discussed as either a single-payer system or a “fill the gaps” approach to the current system. No clear pattern was observed between participants’ views on the feasibility of an essential medicines list and links to or preferences for a particular pharmacare model. A few respondents referred to an essential medicines list as a stepping stone or “political tool” (CSO1) to implement universal single-payer pharmacare.

Most participants, particularly those from civil society, voiced concerns around the influence of groups with vested interests, such as patient groups funded by pharmaceutical industry. Several provincial and territorial government respondents noted industry’s attempts to “consistently and continuously” (PT5) influence policy at the provincial and territorial government level. Some noted the importance of instituting “a perpetual [government] commitment that survives changes in the political cycle*”* (CSO8) at federal, provincial and territorial government levels. Engagement of patients and the public to provide meaningful and deliberate input into processes was deemed important by most, and this engagement was often seen as a method to increase transparency. Participants emphasized the need to manage conflicts of interest carefully to ensure that voices heard were representative of all Canadian concerns and needs.

## Interpretation

Decision-makers and key stakeholders in Canada had different and sometimes skeptical views on the suitability of an essential medicines list in Canada. Nonetheless, there was consensus on 3 important factors that would need to feature in the policy process of a possible approach to an essential medicines list: an independent decision-making body, selection criteria to list medications based primarily on clinical and cost-effectiveness, and clear communication with the public on the purpose and evidence-based focus of the essential medicines list.

A lack of shared understanding of the concept of the essential medicines list and diverse opinions on what constituted an “essential medicine,” based either on what is considered essential for the population or for the individual, were also found in a similar study in Australia, raising questions about the perceptions about essential medicines lists in high-income countries.^22^ A study of key elements of Sweden’s Wise List highlighted 3 of the same process factors identified in this study: comprehensive communication and branding of the list, an independent decision-making body, and strict medicine-selection criteria (along with audience-targeted Wise List editions). The Swedish list was first implemented in 2000 and is widely accepted by physicians and the public.^16^,^17^ Branding a Canadian essential medicines list might be important given some negative associations with the term, and Sweden’s Wise List could be an example to emulate. Similar to the need for an independent body to develop and manage a possible essential medicines list that emerged in this study, other reports have recommended the creation of an arm’s-length body as a key component of pharmacare infrastructure.^7^,^32^ The Federal Advisory Council on the Implementation of National Pharmacare’s recent recommendations for universal, single-payer public pharmacare to be implemented through federal leadership, in partnership with provinces and territories, also include a “Canadian drug agency” and suggest that implementation of a national formulary should begin with coverage for a list of essential medicines.^10^ Furthermore, trusted institutions and processes currently exist across public formularies and through bodies such as the Canadian Agency for Drugs and Technologies in Health, which can be used to develop an essential medicines list or national formulary.

### Limitations

As with most qualitative research, we cannot claim that our findings are representative of all stakeholder views or generalizable beyond our study population. For example, it is possible that non-respondents may hold negative views on an essential medicines list. However, many decision-makers who declined to participate did so because of perceived conflicts of interest in the context of their concurrent contributions to the consultations of the Federal Advisory Council on the Implementation of National Pharmacare in 2018, which ultimately recommended a list of essential medicines as an intermediary for a comprehensive national formulary.^10^ We did not directly interview patients, caregivers or community members, who represent important voices in this context, although public and prescriber perceptions on an essential medicines list have been captured to some extent elsewhere.^33^,^34^

Some variations in data-collection methods may have affected the findings. First, for study feasibility, the interview setting was primarily over telephone; although this is a valid method to collect interview data,^35^ it may have affected rapport and candor compared with in-person interviews. Careful attention was given to establish rapport in each interview.^27^ Second, field notes data from the 3 interviews that were not audio-recorded may have lacked rich descriptions present in transcript data, which may have resulted in some codes or themes being missed. Detailed field notes were taken during and after these interviews to capture responses as best possible.^27^

### Conclusion

Although stakeholders’ views on the suitability and content of a Canadian essential medicines list varied, there was consensus on the process to formulate and implement such a list or common national formulary. The concept of selecting priority medicines based on the best evidence appeared to be widely supported but probably requires careful communication with diverse stakeholders. Further work is needed to establish understanding of how patients, clinicians and the public perceive the concept of a Canadian essential medicines list.
